# Evolution of Tumor Clones in Adult Acute Lymphoblastic Leukemia

**Published:** 2016

**Authors:** S. Yu. Smirnova, Yu. V. Sidorova, N. V. Ryzhikova, K. A. Sychevskaya, E. N. Parovichnikova, A. B. Sudarikov

**Affiliations:** National Hematology Research Center, Novy Zykovskiy Proezd, 4a, Moscow, 125167, Russia; Faculty of Basic Medicine, Lomonosov Moscow State University, Lomonosov Ave., 31/5, Moscow, 119192, Russia

**Keywords:** acute lymphoblastic leukemia, relapse, IG and TCR gene rearrangements

## Abstract

Clonal instability of a tumor cell population in acute lymphoblastic leukemia (ALL) may complicate the monitoring of a minimal residual disease (MRD) by means of patient-specific targets identified at the disease onset. Most of the data concerning the possible instability of rearranged clonal *TCR *and *IG *genes during disease recurrence were obtained for ALL in children. The appropriate features of adult ALL, which are known to differ from those of childhood ALL in certain biological characteristics and prognosis, remain insufficiently studied. The aim of this study was to assess the stability of *IG *and *TCR *gene rearrangements in adult ALL. Rearrangements were identified according to the BIOMED-2 protocol (PCR followed by fragment analysis). Mismatch in clonal rearrangements at onset and relapse was identified in 83% of patients, indicating clonal instability during treatment. Clonal evolution and diversity of *IG *and *TCR *gene rearrangements may be one of the tumor progression mechanisms. New rearrangements may emerge due to residual VDJ-recombinase activity in tumor cells. Also, many clonal *IG *and *TCR *gene rearrangements may be present at different levels at a diagnosis, but less abundant clones may be “invisible” due to limited detection sensitivity. Later, major clones may disappear in the course of chemotherapy, while others may proliferate. Investigation of clonal evolution and heterogeneity in ALL and their impact on the treatment efficacy will contribute to the identification of new prognostic factors and the development of therapeutic approaches.

## INTRODUCTION

Acute leukemias are a heterogeneous group of neoplastic diseases of the hematopoietic tissue, which are characterized by overproduction and accumulation of morphologically immature (blast) hematopoietic cells in the bone marrow. Depending on the hematopoietic lineage giving rise to tumor cells, acute leukemias are conventionally divided into acute lymphoblastic and acute myeloid leukemias. Untreated, the disease rapidly progresses and always results in the death of the patient. The most common causes of death are severe infectious and hemorrhagic complications arising from the replacement of normal hematopoietic tissue with blast cells.

The central goal of a treatment for any type of leukemia is the eradication of the tumor clone, restoration of normal hematopoiesis, and the achievement of long-term relapse-free survival of patients. The introduction of cytostatic drugs in clinical practice in the late 1960s enabled the achievement of complete remission in 85–95% of children with ALL [1]. In that case, an important prognostic factor is age; event-free survival of children in various age groups varies from 83–97% (1–5 years) to 49–66% (10–15 years). Recently, the Russian Research Group for the Treatment of Acute Lymphoblastic Leukemia (RALL) has demonstrated that the 5-year relapse-free survival rate in adult patients under 30 years of age is 71.5%, while this indicator in patients aged 30–55 years is lower – 61.8% [2]. Adults and children with ALL have been demonstrated to differ not only in the survival rate, but also in the biological properties and prognosis of the disease [3, 4]. In particular, a favorable prognostic group in adults includes the T cell type of the disease, whereas this type in children is considered prognostically adverse. In addition, adults are more likely to show prognostically adverse chromosomal aberrations (t(9;22), t(4;11)), myeloid antigens on the membrane of tumor cells, and hyperleukocytosis at the disease onset, and they are more often diagnosed with the T-cell immunophenotype [3, 4].

Another important factor that affects the prognosis in ALL is the residual amount of tumor cells in the bone marrow, or minimal residual disease (MRD). MRD evaluation is considered not only as an independent prognostic factor, but also as a criterion for allocating patients into relapse risk groups [5-7]. The most suitable for MRD quantification are techniques with the highest sensitivity level (10–4–10–5), such as real-time PCR (RT-PCR) with patient-specific primers, as well as multicolor flow cytometry. MRD evaluation in ALL patients by PCR is based on the identification of the clonal rearrangements of the T-cell receptor (TCR) and the immunoglobulin (IG) genes in the tumor cells, and the selection of patient-specific primers to the CDR3- region of the genes [8].

Clonal rearrangements of the *IG *and *TCR *genes occur in 98% of B-ALL patients and in 95% of T-ALL patients [9]. Because different chromosomal aberrations are found in tumor cells derived from different patients, only rearranged *IG *and *TCR *genes are considered to be universal markers for monitoring tumor clones in almost all patients during disease/therapy. Detection of clonality alone is not enough for ALL diagnosis. Clonal rearrangements are sometimes found also in the reactive (non-tumor) processes of inflammatory, infectious, or autoimmune genesis. A clonal product in these cases is usually detected on a polyclonal background. The differential diagnosis between tumor and non-tumor lymphoproliferation is somewhat difficult in some lymphomas, mycosis fungoides, and Sezary syndrome; however, a study of clonality in ALL when most peripheral blood lymphocytes (> 20%) are represented by tumor cells is not associated with these difficulties.

RT-PCR with patient-specific primers selected for a unique nucleotide sequence of the V-D-J-region of clonally rearranged *IG *or *TCR *genes enables a highly sensitive (10–4–10–5) evaluation of the amount of residual tumor cells in ALL patients [10]. However, the data obtained from studying clonal rearrangements at the onset and relapse of ALL in children indicate that *IG *and *TCR *gene rearrangements can change during the disease: part of the identified clonal rearrangements disappears at relapse, and/or new rearrangements emerge. It should be noted that we are talking just about a partial change in clonal rearrangements, because a complete change in *IG *and *TCR *gene rearrangements at relapse indicates a development of secondary ALL [11, 12]. Partial differences in clonal rearrangements at the onset and relapse occur in 67–70% of children with B-ALL and in 45–50% of children with T-ALL [13-15]. Data on the evolution of tumor clones in adult ALL is scant [11]. Szczepanski et al. reported on an evaluation of *TCR *genes in 9 adults with T-ALL [11]. The overall stability of *TCR *genes in the adult T-ALL was shown to be higher (97%) than that in childhood T-ALL (86%) [11]. However, *IG *gene rearrangements at the onset and relapse were not studied.

Alteration of clonal rearrangements, i.e. clonal evolution of the tumor, may lead to a loss of the target for MRD studies and to false negative results. Therefore, the suitability of a particular rearrangement to study MDR in ALL is determined not only by the rearrangement detection frequency, but also by its stability. The crucial data on the stability and frequency of various rearrangements in B-ALL and T-ALL are summarized in *Table 1* [5-11, 15-19].

**Table 1:** Stability and detection the rate of clonal rearrangements in B-ALL and T-ALL [7]

Gene	Rearrangement	B-ALL				T-ALL	
		Rate, %		Stability, %		Rate, %	Stability, %
		mono	oligo	mono	oligo		
IGH	VH-JH (complete)	93	30-40	88	47	5	NT
	DH-JH (incomplete)	20	50-60	57	38	23	NT
	All IGH	98	40	85	44	23	NT
IGK	Vκ-Kde	45	5-10	95	40	0	NA
	Intron RSS-Kde	25	5-10	95	40	0	NA
	All Kde	50	5-10	95	40	0	NA
TCRB	VB-JB (complete)	21	10-15	89	60	77	79
	DB-JB (incomplete)	14	10-15	67	0	55	80
	All TCRB	33	10-15	81	43	92	80
TCRG	VG-JG	55	15	75		95	86
TCRD	VD-JD or DD-JD1	< 1	NA	NA	NA	50	100
	VD2-DD3 or DD2-DD3	40	20-25	86	26	55	100
	All TCRD	40%	20–25%	86%	26%	55%	100%

Note.

NT – not tested.

NA – not applicable.

TCR δ-chain (TCRD) gene rearrangements are specific to early stages of T cells development and occur in about 55% of T-ALL cases only [20]. TCR γ-chain (TCRG) gene rearrangements occur in 95% of T-ALL patients [21]; TCR β-chain (TCRB) gene rearrangements occur in 92% of T-ALL patients. The stability of TCRB rearrangements in T-ALL relapses in children was shown to be lower than that of γ- and δ-chains – 80, 86, and 100%, respectively (*Table 1*) [11]. Despite a high detection rate and high stability, monoclonal rearrangements of γ-chain genes are not the best target for MRD monitoring, because they possess a short fragment of inserted nucleotides [22]. According to the published data, T-ALL is often more resistant to therapy and, therefore, MRD positive than B-ALL [23]. A high stability of *IGK *gene rearrangements (95%) in B-ALL children, complete V-D-J-rearrangements of the *IGH *(88%), *TCRB *(89%), and *TCRD *(86%) genes, a relatively high stability of *TCRG *gene rearrangements (75%), and a low stability of incomplete (D-J) rearrangements of *IGH *genes (57%) and incomplete rearrangements of *TCRB *genes (67%) were established (*Table 1*). Furthermore, oligoclonal rearrangements were initially detected in a large proportion of childhood B-ALL cases (26–30%) [13-15]. Clonal products of an incomplete gene rearrangement and derived clonal products with complete rearrangements can be present in ALL, which is explained by the action of V(D)J-recombinases and the ongoing process of immunoglobulin and *TCR *gene rearrangements in early progenitor cells [11, 15, 24]. Oligoclonality (presence of two or more clones) is most often detected in *IGH *genes: complete rearrangements – in 30–40% of cases, incomplete rearrangements – in 50–60% of cases, and TCR δ-chain gene rearrangements – in 20–25% of cases (*Table 1*). Oligoclonal rearrangements are not recommended for use as a target for MRD evaluation, because they are unstable and often produce false negative results.

The evolution of tumor cells (alteration of clonal *TCR *and *IGH *gene rearrangements) at relapse has been studied mainly in childhood ALL. Data on adult ALL is very limited. Given that adult and childhood ALLs have different biological characteristics and prognosis, the aim of our study was to examine patterns of clonal immunoglobulin and T-cell receptor gene rearrangements and how stable they are in adults with B-ALL and T-ALL who had undergone treatment at the Hematology Research Center.

## MATERIALS AND METHODS

**Patients and samples**

**Table 2:** Brief characteristics of ALL patients

Age, years	19–59 (M, 28)
Gender, M/F	32/31
B-ALL/T-ALL/biphenotypic ALL	34/28/1
Number of relapses (B-ALL/T-ALL)	6 (4/2)
Relapse-free time, months	5.4–11.6 (M, 6.2)

Note.

M – median age.

The study included 63 ALL patients: 34 patients with B-cell ALL, including two patients with Ph+ ALL; 28 patients with T-cell ALL; and one patient with biphenotypic ALL (*Table 2*). All patients underwent a standard cytogenetic examination and a FISH-study of bone marrow cells using fluorescent probes t(9;22) and t(4;11) (*Table 3*). Out of the 63 patients, 20 had a normal karyotype, 17 had no mitosis, and six had different variations of chromosome 9 and/or 22. The translocation t(9;22) was detected by FISH, and the chimeric transcript BCR/ABL (p190) was identified by a molecular-genetic method (Ph+ B-ALL). In five patients, the translocation t(4; 11) was identified by FISH and the chimeric transcript MLL-EPS15 was detected by PCR. Multiple chromosomal abnormalities were found in seven patients; four patients had trisomy 21. We studied DNA from all 63 samples of bone marrow at the disease onset. The patients’ age ranged from 19 to 59 years (median, 28 years). In 6 of the 63 patients, clonal rearrangements were studied at the onset and relapse. The time to relapse ranged from 5.4 to 11.6 months. The patients were observed at the Department of Chemotherapy for Hemoblastoses and Hematopoiesis Depressions of the Hematology Research Center (HRC). The diagnosis was made according to the WHO classification. All the patients enrolled in the study provided their consent to data processing. Blood from healthy donors was obtained at the HRC blood transfusion department.

**Table 3:** Results of conventional cytogenetic testing and FISH-analysis of translocations t(4;11) and t(9;22) in ALL patients

Patient	ALL type	conventional cytogenetic testing, FISH, PCR
1	B-II	No mitosis
2	B-I	Normal karyotype
3	B-I	der(7)add(p22), –8?, der(9), i(q10), der(14), add(q32?), +mar der(9)?(17)cp/46, XX [3]
4	B-I	Additional material on the short arm of chromosome 10; trisomy of chromosomes X, 12, and 22; FISH t(4;11); MLL-EPS15 identified by PCR
5	B-I	55XX; derivatives of chromosomes 3 and 11; deletion of the short arm of chromosome 12 and the long arm of chromosome 13
6	B-I	Normal karyotype
7	B-I	Normal karyotype
8	B-I	No mitosis, FISH t(4;11), MLL-EPS15 identified by PCR
9	B-II	Additional signal from an IGH gene locus (14q32) was identified in 80% of nuclei (trisomy of chromosome 14? another translocation involving an IGH gene locus)
10	B-II	Normal karyotype
11	B-II	No mitosis
12	B-II	53XY? +X,+4,+6,+14,+21,+21,+mar [10]
13	B-II	Normal karyotype
14	B-II	No mitosis
15	B-II	No mitosis
16	B-II	In 15%, two additional signals each from loci of ABL (9q34) and BCR (22q11) genes, tetrasomy of chromosomes 9 and 22?
17	B-II	Trisomy 21
18	B-II	Normal karyotype
19	B-II	Trisomy 21
20	B-II	Trisomy 21, monosomy 13
21	B-II	Normal karyotype
22	B-II	No mitosis
23	B-II	Normal karyotype
24	B-II	Normal karyotype
25	B-II	Normal karyotype
26	B-III	Two cells with del (11), FISH t(4;11), MLL-EPS15 identified by PCR
27	B-III	Normal karyotype
28	B-III	47XX +5 (5q31)
29	B-III	54X, ?+X, Y, +4, +5, +6, ?–7, +14, +21, +22, +?mar or i(7)(q10) or i(8)(q10), +mar[19], 46XY
30	B-III	+8+11+21
31	B-Ph+	No mitosis, FISH t(9;22), BCR-ABL identified by PCR
32	B-Ph+	Normal karyotype, FISH t(9;22), BCR-ABL identified by PCR
33	B-II	No mitosis
34	B-II	No mitosis
35	T-I	46XY[2]/90-92, XXYY, =mar[10]
36	T-I	No mitosis
37	T-I	11q23 rearranged, FISH t(4;11), MLL-EPS15 identified by PCR
38	T-I	Normal karyotype
39	T-I	del9(p13)
40	T-I	No mitosis
41	T-I	Normal karyotype
42	T-I	Trisomy in 15.5%; in 45% tetrasomy in the gene locus PMLL\11q23; FISH t(4;11); MLL-EPS15 identified by PCR
43	T-I	Normal karyotype
44	T-II	Normal karyotype
45	T-II	Normal karyotype
46	T-II	Normal karyotype
47	T-II	No mitosis
48	T-II	Deletion of the long arm of chromosome 5? or translocation t(5;?), derivatives of chromosomes 2, 4, 5, 7, 22, and 17 (with involvement of the p53 gene)
49	T-II	Trisomy of chromosome 8
50	T-II	(47, XY, +8 (20))
51	T-II	Normal karyotype
52	T-II	No mitosis
53	T-III	Normal karyotype
54	T-III	47, XY+mar [20]
55	T-III	der(1)add(p36)?dup(p31p36)?{20}; momosomy 9 or deletion of locus 9q34
56	T-III	No mitosis
57	T-III	Derivative of chromosome 11, deletion of the long arm of chromosome 6
58	T-III	No mitosis
59	T-III	No mitosis
60	T-III	No mitosis
61	T-IV	t(6;17) +20
62	T-I	del 11q23
63	Biphenotypic ALL	47, XY, der(2)add(p24-25), +5, del(7)(q22), del(13)(q11-q34), +14[6]/46, XY[4]

^#^The structure was solved by NMR in contrast to the other structures solved by X-ray crystallography.

**Analysis of clonality using IG/TCR gene rearrangements**

Leukocytes and DNA were isolated from peripheral blood as described previously [25]. The DNA concentration was determined spectrophotometrically. DNA samples were stored at –20 °C. B- and T-cell clonality was determined using multiplex BIOMED-2 primer sets for fragment analysis [26]. B-cell clonality was evaluated by IGH heavy chain (VH-JH FR1/FR2/ FR3/DH-JH) and IGK κ-light chain (Vk-Jk/Vk-KDE/ IntronRSS-KDE) gene rearrangements. T-cell clonality was evaluated by gene rearrangements of the T-cell receptors TCRG (VG-JG), TCRB (VB-JB/DB-JB), and TCRD (VD-JD/DD2-JD/VD-DD3/DD2-DD3). All *IG *and *TCR *gene loci were analyzed in multiplex reactions with a large number of primers clustered in several tubes according to the BIOMED-2 protocol recommendations (briefly described in *Table 4*). The *TCRB *genes were amplified using a TCRB Gene Clonality Assay ABI Fluorescence Detection kit (Invivoscribe Technologies, USA) according to the manufacturer’s recommendations. A mixture (25 μL) for the PCR of the *IGH*, *IGK*, *TCRG*, and *TCRD *genes contained 5 pM of each primer (Synthol, Russia), 100–200 ng of DNA, and 12.5 μL of 2 × PCRMasterMix (Promega, USA). Amplification was performed on a DNAEngine automated thermocycler (BioRad, USA). PCR conditions were as follows: 95 °C (7 min), then 35 cycles of 95 °C (45 s), 60 °C (45 s), 72 °C (45 s), and 72 °C (10 min). The cell lines Jurkat and Daudi were used as a positive (clonal) control. Peripheral blood mononuclear cells of healthy donors were used as a polyclonal control. A fragment analysis of PCR products was performed on an ABIPRISM 3130 Genetic Analyzer (Applied Biosystems, USA). For this purpose, 2 μL of a 20-fold diluted PCR product was mixed with 10 μL of Hi-Di formamide (Applied Biosystems, USA) and 0.04 μL of a GeneScan 500-LIS Size Standard (Applied Biosystems, USA). After denaturation at 95 °C for 3 min and subsequent cooling, 10 μL of the mixture was added to a well of a 96-well plate and high resolution capillary electrophoresis was performed on a POP-4 polymer (Applied Biosystems, USA). The fluorescence of amplicons and their profile were evaluated using the GeneMapper v.4.0 software (Applied Biosystems, USA).

**Table 4:** Description of multiplex reactions and PCR primers according to the BIOMED-2 protocol

Gene	Primer set	Forward primers	Reverse primers (labeled)	Product length, bp
IGH	A	VH1-7 (FR1)	JHcons FAM	310–360
	B	VH1-7 (FR2)	JHcons FAM	250–295
	C	VH1-7 (FR3)	JHcons FAM	100–170
	E	DH1-6	JHcons TAMRA	110–290 and 390–420
	D	DH7	JHcons TAMRA	100–130
IGK	A	Vκ1/6-7	Jκ1-4, Jκ5 FAM	120–300
	B	Vκ1/6-7, INTR	KDE-FAM	210–390
TCRD	D1	Dδ2,Vδ1-Vδ6	Jδ1FAM, Jδ2R6G Jδ3TAMRA, Jδ4ROX	120–280
	D2	Dδ2,Vδ1-Vδ6	Dδ3FAM	130–280
TCRG	GA	Vγ1f, Vγ10	Jγ1/2FAM, Jp1/2 R6G	145–255
	GB	Vγ9, Vγ11	Jγ1/2FAM, Jp1/2 R6G	80–220
TCRB	A	Vβ2-Vβ24	Jβ1.1, Jβ1.6HEX Jβ2.2, Jβ2.6, Jβ2.7FAM	240–285
	B	Vβ2-Vβ24	Jβ2.1, Jβ2.3, Jβ2.4, Jβ2.5 FAM	240–285
	C	Dβ1, Dβ2	Jβ1.1, Jβ1.6HEX Jβ2.1, Jβ2.7FAM	170–210 285–325

## RESULTS AND DISCUSSION

Clonal rearrangements were studied in 34 patients with B-cell ALL, 28 patients with T-cell ALL, and 1 patient with biphenotypic ALL. The frequencies of clonal TCR γ-, β-, and δ-chain and IG heavy- and light-chain gene rearrangements in B- and T-ALL are presented in *Table 5*. A biallelic rearrangement (two peaks) of *TCRG *genes and an oligoclonal rearrangement (four peaks) of *TCRD *genes were detected in the patient with biphenotypic ALL. In patients with B-cell ALL, the IG heavy-chain (82.4%) and TCR γ-chain (76.5%) gene rearrangements were the most frequent; TCR β-chain and IG κ-chain gene rearrangements were found in 38.2% of cases; TCR δ-chain gene rearrangements occurred in 55.9% of cases. In patients with T-cell ALL, TCR γ-, δ-, and β-chain gene rearrangements were detected in 89.3%, 64.3%, and 60.7% of cases, respectively. *IGH *rearrangements in T-ALL occurred less often (28.6%) than others. A Vk/KDE rearrangement of immunoglobulin κ-light chain genes was found in one case of T-ALL. Our data on the frequency of clonal *IG *and *TCR *gene rearrangements somewhat differ from the data obtained in international studies, which may be associated with our small sample size. Oligoclonal rearrangements (three or more clonal peaks) occurred both in B-ALL (*IGH *in 12% (4 of 34) of patients, *TCRD *in 18% (6 of 34) of patients) and in T-ALL (*TCRD *in 32% (9 of 28) of patients).

**Table 5:** Detection rate (%) of clonal TCR γ-, β-, and δ-chain gene rearrangements and IG heavy- and light-chain gene rearrangements in B- and T-ALL

Rearrangements		B-ALL (n = 34)	T-ALL (n = 28)
TCRG	VG–JG	74.3 (n = 26)	89.3 (n = 25)
TCRB	VB–JB (complete)	26.5 (n = 9)	50 (n = 14)
	DB–JB (incomplete)	23.5 (n = 8)	46.4 (n = 13)
	All TCRB	38.2 (n = 13)	60.7 (n = 17)
TCRD	VD–JD/DD2–JD	17.6 (n = 6)	53.6 (n = 15)
	VD–DD3/DD2–DD3	47.1 (n = 16)	32.1 (n = 9)
	All TCRD	55.9 (n = 19)	64.3 (n = 18)
IGH	VH–JHFR1/FR2/FR3 (complete)	73.5 (n = 25)	7.1 (n = 2)
	DH–JH (incomplete)	26.5 (n = 9)	25 (n = 7)
	All IGH	82.4 (n = 28)	28.6 (n = 8)
IGK	Vk–Jk	26.5 (n = 9)	0 (n = 0)
	Vk–KDE/Intron RSS–KDE	26.5 (n = 9)	3.6 (n = 1)
	All IGK	38.2 (n = 13)	3.6 (n = 1)

In six patients, clonal rearrangements were investigated at the onset and relapse. A total of 17 clonal *TCR *and 5 clonal *IG *gene rearrangements were identified at the onset. Six clonal *TCR *and three clonal *IG *gene rearrangements different from those identified at the onset were detected at relapse (*Table 6*).

**Table 6:** Clonal products identified at the onset and relapse in six patients diagnosed with ALL

Patient/ diagnosis	Case 1 T-ALL		Case 2 T-ALL		Case 3 B-ALL		Case 4 B-ALL		Case 5 B-ALL		Case 6 B-ALL	
	O	R	O	R	O	R	O	R	O	R	O	R
TCRG-GA	+	+	+	+	-	-	+	-	-	+	+	+
TCRG-GB	+	+	-	-	-	-	+	-	-	+	-	+
TCRB-A	+	+	-	-	-	-	+	-	-	-	-	+
TCRB-B	-	-	-	-	-	-	-	-	-	-	+	+
TCRB-C	-	-	-	-	-	-	-	-	-	-	-	-
TCRD-D1	+	+	+	+1	+	+	+	+	+	+	-	-
TCRD-D2	+	+	-	-	-	+	+	-	+	-	-	-
IGH-A/IGH-B/IGH-C	-	-	-	-	-	+	-	-	+	+1	+	+
VK-A	-	-	-	-	-	+	+	-	-	-	+	-
VK-B	-	-	-	-	-	-	+	+	-	-	-	-

Note.

“+” – monoclonal rearrangement,

“–” – polyclonal rearrangement,

“+1” – initial clonal rearrangement is detected along with an additional rearrangement different from the one identified at the onset,

O – onset,

R – relapse.

**Fig. 1:**
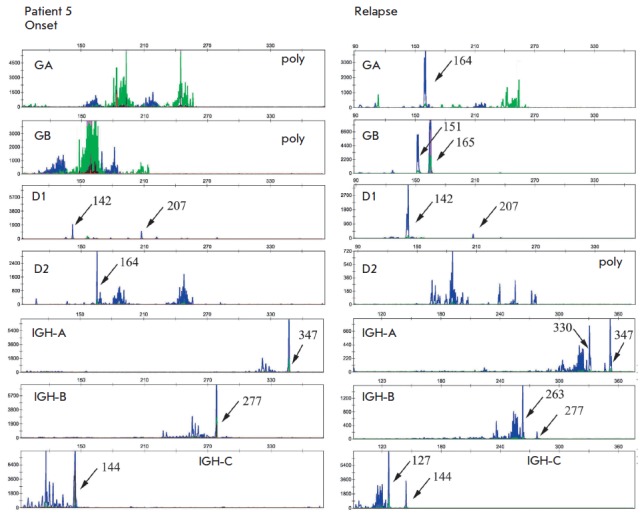
Fragment analysis of *TCR*, *TCD*, and *IGH *gene amplification products in patient 5 at the onset and relapse. Two 142 and 207 bp clonal products (indicated by arrows) of *TCRD *gene rearrangements and a 347 bp clonal product of *IGH *gene rearrangements were amplified at the disease onset. In this patient, the rearrangements remained at the relapse and new clonal products were also identified (*TCRGA *– 164 bp, *TCRGB *– 151 and 165 bp; *IGHA *– 330 bp, *IGHB *– 263 bp, *IGHC *– 127 bp).

**Fig. 2:**
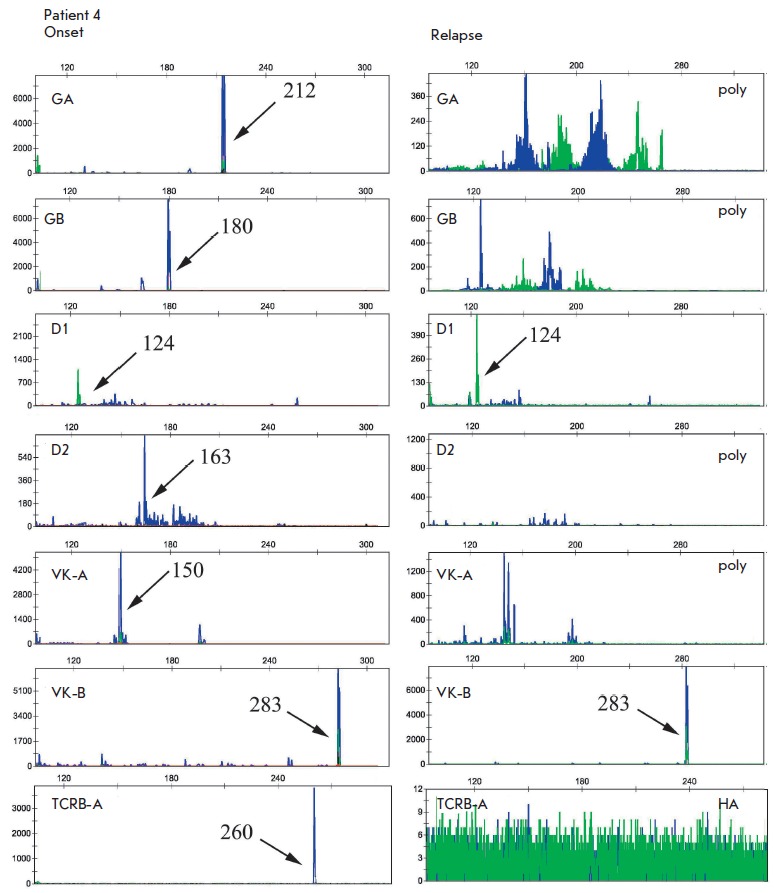
Fragment analysis of *TCRG*, *TCRD*, *IGK*, and *TCRB *gene amplification products in patient 4 at the onset and relapse. Seven clonal rearrangements were identified at the diagnosis. Only two of them were preserved at the relapse (*TCRD *– 124 bp; *IGK *– 283 bp).

Two patients with B-cell ALL had a loss of one of the clonal rearrangements identified at the onset, with new rearrangements simultaneously emerging (patient 5 in *Fig. 1* and patient 6 in *Table 6*). In one patient diagnosed with early precursor T-ALL, the clonal TCR γ-, β-, and δ-chain gene rearrangements completely coincided at the onset and relapse (case 1). In one T-ALL patient, new rearrangements emerged, in addition to the clonal rearrangements identified at the onset (case 2). In one patient, only two of the seven rearrangements identified at the onset were preserved up to the relapse (*Fig. 2*, patient 4). In one patient, only one clonal TCR δ-chain gene rearrangement was detected at the B-ALL onset, which was preserved up to the relapse, but several new rearrangements emerged, including clonal *IGH*and IG light κ-chain gene rearrangements.

We were able to demonstrate that at least one of the initially detected clonal products was preserved up to ALL relapse in all patients (*Table 6*). This confirms the data showing that at least one initial clonal product is preserved even in the case of late ALL relapse in children (more than 5 years after remission achievement) [27]. In our work, differences in clonal rearrangements at the onset and relapse were found in five out of six (83%) patients. Even with this small sample size, we observed clonal evolution at relapse, which raises the issue of initial selection of the target for MRD quantification. At least two independent targets with high stability are conventionally used to minimize the risk of false positive results. However, in practice, a patient-specific primer with the desired specificity and sensitivity cannot be selected for every target. First of all, this applies to incomplete rearrangements or gene rearrangements lacking the D-segment, e.g. *TCRG *(Vγ-Jγ). We found a loss of the patient-specific targets identified in three patients at the onset. To trace minor subclones at the onset and to evaluate their behavior in the presence of therapy, we decided to increase the initial sensitivity of the method. V- and J-family specific primers were used to re-examine the initial material for the presence of the clones that emerged at the relapse. The use of these primers increases the sensitivity of tumor cell detection from 10–1 to 10–2–10–3. However, even with this sensitivity, subclones were not detected at the onset, which suggests a small size of the subclones and confirms the data of other studies. For example, in 77% (35 of 45) of childhood B-ALL cases, clones with new rearrangements at relapse were present only as small subclones at the onset [28]. The size of these resistant subclones ranged from 10–2 to 10–5 cells, and the lower the cell number was, the longer the time to relapse was [29].

Recently, acute lymphoblastic leukemias were shown to have a complex and genetically heterogeneous composition of tumor cells within one disease [30, 31]. In most ALL cases, clonal evolution is based on the reactivation of one of the minor subclones, which is resistant to therapy [29, 32, 33]. Clonal diversity is the mechanism underlying tumor progression. Some clonal cells are likely to have properties that are different from those of other cells (genetic mutations, division rate, immunological maturity), making them resistant to chemotherapy. The causes behind late reactivation of the initial tumor clone remain unknown. Perhaps, the immune surveillance and the mechanisms of antitumor immunity weaken, or new genetic mutations emerge in tumor cells that are then reactivated. The use of quantitative methods to evaluate MRD is an independent prognostic factor and a criterion for the stratification of patients into relapse-risk groups. The spectrum of clonal rearrangements can vary during the disease. This process can occur during early induction therapy, which leads to false negative results of MRD evaluation and prevents a stratification of patients into risk groups. Successful monitoring of the minimal residual disease can be ensured only through the selection of patient-specific primers for each clonal target identified at the onset.

## CONCLUSION

Five out of six (83%) patients studied had differences in clonal rearrangements at the onset and relapse, which indicates clonal instability in the presence of polychemotherapy. Tumor cells in ALL initially show a complex and genetically heterogeneous composition; while some clones disappear due to polychemotherapy, others that are unidentified because of the insufficient sensitivity of the method acquire the ability to activate. Clonal evolution is one of the mechanisms behind tumor progression and is a serious obstacle to the quantification of MRD by PCR. We have demonstrated that the absence of amplification with patient-specific primers selected for targets sequenced at the disease onset cannot fully guarantee an absence of residual disease because tumor clones were shown to be unstable in some cases of acute leukemia. Therefore, clonality should be re-examined in doubtful cases of suspected relapse and the absence of amplification with patient-specific primers. Investigation of clonal evolution mechanisms and the ability of chemotherapy to affect clonal evolution processes will contribute to the development of new prognostic factors and therapeutic approaches.

## Abbreviations

ALL: acute lymphoblastic leukemia
MRD: minimal residual disease
RT-PCR: real-time PCR
TCR: T cell receptor
TCRG: T cell receptor γ
TCRB: T cell receptor β
TCRD: T cell receptor δ
IG: immunoglobulin
IGH: immunoglobulin heavy chain
IGK: light chain κ
